# Socioeconomic status and survival outcomes in elderly cancer patients: A national health insurance service‐elderly sample cohort study

**DOI:** 10.1002/cam4.2231

**Published:** 2019-05-08

**Authors:** Bum‐Sup Jang, Ji Hyun Chang

**Affiliations:** ^1^ Department of Radiation Oncology Seoul National University Bundang Hospital Seoul South Korea; ^2^ Department of Radiation Oncology SMG-SNU Boramae Medical Center Seoul South Korea

**Keywords:** cancer mortality, elderly, income, regional deprivation, Socioeconomic status

## Abstract

**Background:**

We hypothesized that lower socioeconomic status (SES) was associated with higher all‐cause mortality in patients newly diagnosed with cancer, particularly in the elderly population.

**Methods:**

We collected study patients from the stratified random sample of Korean National Health Insurance Elderly Cohort (2002‐2015). The Cox's proportional hazards model was used to investigate the risk factors for mortality. Income level and composite deprivation index (CDI) 2010 were used to define the SES: low, intermediate, and high SES groups. The comorbidities were measured using Charlson Comorbidity Index score. After a wash‐out period (2002), the final study population was 108 626 (2003‐2015).

**Results:**

In multivariate analysis, low SES was associated with poor overall survival (OS) (HR = 1.08, 95% CI: 1.05‐1.12, *P* < 0.001) and cancer‐specific survival (CSS) (HR = 1.11, 95% CI: 1.06‐1.16, *P* < 0.001) particularly for patients aged 70‐79 years. High SES was favorable prognostic factor of OS in patients aged 60‐69 years (HR = 0.85, 95% CI: 0.81‐0.89, *P* < 0.001), 70‐79 years (HR = 0.90, 95% CI: 0.87‐0.93, *P* < 0.001), and ≥80 years (HR = 0.91, 95% CI: 0.87‐0.96, *P* < 0.001). However, SES was not associated with CSS in advanced age patients (≥80 years). Patients with low SES manifesting colorectal, urinary, liver, gastric, melanoma, and esophageal cancers demonstrated worse OS, compared to patients with intermediate SES. Also, low SES patients with urinary, liver, or colorectal cancers or melanoma demonstrated worse CSS compared to those with intermediate SES.

**Conclusion:**

Low SES at the time of cancer diagnosis is associated with increased risk of OS and CSS in elderly patients. Depending on cancer sites, different patterns of OS and CSS were observed according to SES. Further elucidation of the causes underlying these phenomena is needed along with appropriate support for elderly cancer patients with low SES.

## 1 INTRODUCTION

Cancer is one of the public health concerns in Korea due to its status as the leading cause of death in Korea. Newly diagnosed cancers are expected to total 204 999 and 82 155 cancer deaths in 2018, with increasing burden due to the aging of population.^1^ In particular, the additional economic burden has increased 1.8‐fold during 2000‐2010.^2^

A growing body of evidence suggests that patients' socioeconomic status (SES) plays an independent role in oncologic outcomes^3^ in addition to cancer biology. It is well established that SES based on individual income level has been linked to cancer mortality.^4^ However, neighborhood poverty or regional deprivation has been considered as an important component of SES affecting the outcomes in non‐small cell lung,^5^ esophageal,^6^ anal cancer.^7^ Income level and regional deprivation has often been separately investigated, but the impact of both combined is still lacking.

Managing elderly patients with cancer is an important issue in oncology when considering treatment decisions. Cancer diagnosis in the elderly is likely to be at an advanced stage, leading to less aggressive or delayed treatment. Furthermore, a lower survival among elderly patients is attributed to disparities among elderly patients^8^ in the United States. The individual and environmental factors associated with cancer development among the elderly patients in Korea have yet to be investigated.

The current study highlights the impact of SES determined by household income level and regional deprivation to clinical outcomes in elderly patients with cancer using data derived from a national database.

## 2 METHODS

### 2.1 Data

Data were obtained from the National Health Insurance Service‐Elderly Sample Cohort (NHIS‐ESC) dataset of Korea, which provides support for researchers investigating elderly patients. Of the 5.5 million elderly people (≥60 years) who maintained medical insurance as of December 2002, about 10% were sampled to construct a cohort database. Information about medical insurance, factors of SES, and the use of medical clinics over a period of 14 years (from 2002 to 2015) was stratified and collected without personal identification. The NHISS intentionally masks disease‐related information such as male/female genital, gynecological, breast, or prostate cancers because they are considered sensitive and private information in Korea, and associated with the risk of patient identification. Thus, we cannot analyze patients with these cancers in the study cohort.

### 2.2 Variables

Variables used in the current study include age (60‐69, 70‐79, and ≥80 years), gender, insurance type (local, workplace, and medical aid), the presence of disability, cancer site, cancer diagnosis year, Charlson Comorbidity Index (CCI) score^9^ (≤1 vs >1), Composite Deprivation Index (CDI)^10^ (low vs high) for Korea, and household income. The cost of medical care in this study was measured as the total number of days of medical treatment per capita as a proportion of the total medical expenses per capita. Medical expenses per capita represent the cost of health insurance in patients treated by a medical institution, which is the sum of the corporate and individual contribution. It is the medical fee based on the total medical expenses charged by the medical institution.

The CCI was calculated from weight‐sum of 1‐6 points assigned by International Classification of Disease, 10th revision (ICD‐10) codes of comorbidities, except cancer. The detailed CCI scoring system was reported previously.^9^ We estimated CCI with comorbidities diagnosed before the date of cancer diagnosis in the study cohort.

On the other hand, the CDI included five social exclusions including unemployment, poverty, housing, labor, and social network. The CDI in the current study was derived from the 2004‐2006 National Death Registry data, the 2005 Population Census data, and the 2005‐2006 means‐tested benefit recipients' data in Korea. As a deprivation index, the CDI is comparable to Townsend^11^ and Carstairs index^12^ in England, and is a good proxy variable representing rural deprivation in Korea. The CDI has a value ranging between 0 and 500: the lower the value, the lower is the degree of deprivation of the city or district unit. Due to the limited availability of regional data in the NHIS‐ESC cohort, we calculated the CDI values of small districts within cities by geometric averaging to represent a single CDI value in each city. They were matched to the patients' city by the year of cancer diagnosis. Subsequently, patients were divided into two groups (low‐ vs high‐CDI) according to the median value of CDI score.

The average monthly insurance premium estimated by NHISS was used to represent the household income. The health insurance coverage in Korea is classified into three categories namely: medical aid, local, or workplace health insurance. Individuals are eligible for medical aid when the household income is <$600 per month; otherwise, they are covered by local or workplace health insurance. Monthly premiums for health insurance subscribers for workplace health insurance are determined based on monthly salary, and monthly premiums for local health insurance subscribers are based on the income or property of eligible households. In NHIS‐ESC, the patient's insurance status is encoded as follows: 0 for medical aid and 1‐10 for evenly distributed percentiles according to insurance premium. We divided patients into two groups: high (81st‐100th percentile) vs low‐income group (0‐80th percentile and medical aid). Monthly premiums represent the average obtained from each claim of the cancer patient. We, therefore, assumed that the insurance premiums of the family members were the insurance premiums of the households because of unavailable data regarding the number of households.

### 2.3 Definition of socioeconomic status

Socioeconomic status was defined as the combined household income and regional deprivation. Each patient was classified into three levels of SES: low, intermediate, and high. The patients with low SES were defined by residence in the high‐CDI region (deprived) and with low income, and high SES was defined by living in the low‐CDI region (advantageous) and with high income. Patients living in the high‐CDI region and with high income or those living in low‐CDI region and with low income were classified into an intermediate SES group.

### 2.4 Endpoints

The primary endpoint was overall survival (OS), which is calculated from the date of cancer diagnosis until the date of death. The secondary endpoint was cancer‐specific survival (CSS) defined as the length of time from the date of cancer diagnosis until the date of death from cancer, which was identified in the NHIS‐ESC database.

### 2.5 Study population

**Figure 1:**
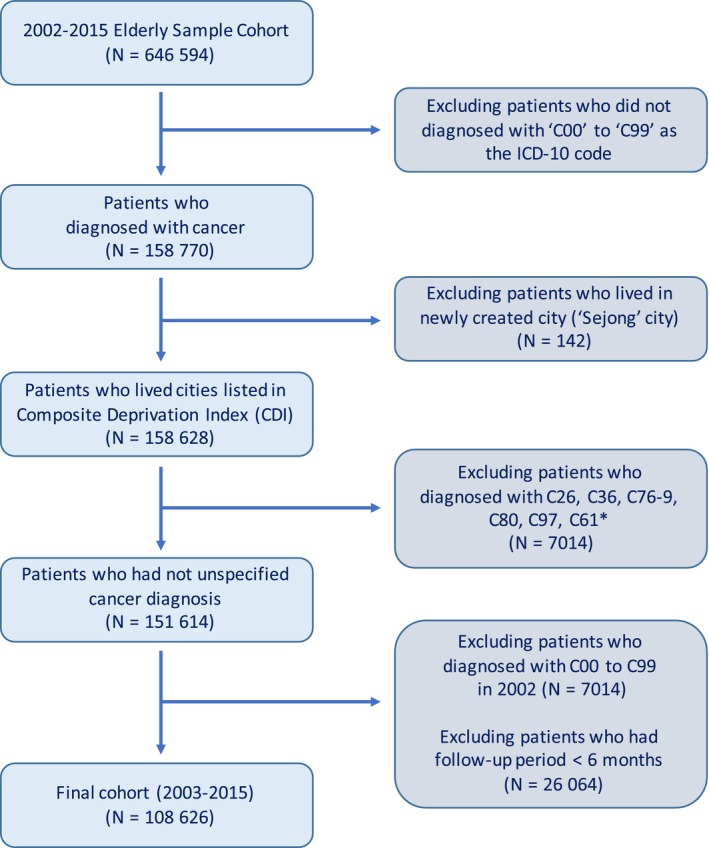
Flow diagram selecting study population. *These ICD‐10 code indicates unspecified neoplasm. ICD‐10, International Classification of Disease, 10th revision (ICD‐10)

The flow diagram for patient selection is summarized in Figure 1. We selected patients with cancer diagnosis from the total NHIS‐ESC database (N = 646 594). To facilitate regional deprivation data such as CDI, we excluded patients who had lived in the newly created city (N = 142), that is “Sejong” city. Also, patients diagnosed with the ICD codes including C26, C36, C76‐9, C80, C97, C61 were excluded (N = 7014) because of their obscure disease status such as other malignant and ill‐defined neoplasms. Subsequently, we imposed a wash‐out period on the study population by excluding patients diagnosed with cancer in 2002 (N = 7014) and those who had a short follow‐up of <6 months (N = 26 064). Finally, we constructed the study cohort including 108 626 elderly cancer patients who were followed up during 13 years (from 2003 to 2015).

### 2.6 Statistical analysis

Baseline characteristics among SES level groups were compared using Chi‐squared test. The Cox proportional hazard models were used to investigate the association between SES and oncologic outcomes in univariate or multivariate analysis. Hazard ratios (HR) with 95% confidence interval (CI) were calculated adjusting for covariates. To verify the assumption of the Cox proportional hazard model, we plotted log‐log plots according to SES after adjustment for diagnosis year, age, insurance type, disability, and CCI scores. The results showed a nearly parallel log‐linear relationship among the SES groups for OS and CSS (Figure S1A,B), supporting the assumptions of the Cox proportional hazard models in the current study. Furthermore, we used robust standard errors to report HR and 95% CI in each Cox proportional hazard model. Kaplan‐Meier methods were used to depict CSS and OS curves according to SES level. A two‐side *P*‐value < 0.05 was considered statistically significant. All statistical methods were performed using STATA/MP 15.1 (Stata Corp) and SAS 9.2 (SAS Institute) provided by NHIS.

## 3 RESULTS

### 3.1 Characteristics

Baseline characteristics comparing high, intermediate, and low SES groups are summarized in Table 1. Significant differences were found in all variables between SES groups: age, gender, insurance type, the presence of disability, CCI score, year of cancer diagnosis, and cancer sites. Patients aged 70‐79 years comprised nearly half of the population in each SES group (*P* < 0.001). Male patients were more significantly distributed in the high SES rather than low or intermediate SES groups (*P* < 0.001). Patients whose CCI score was >1 were predominantly found in the high SES group (74.6%) compared to low (72.0%) or middle SES group (72.7%) (*P* < 0.001). Most cancer was diagnosed from 2006 to 2010. Regarding cancer sites, the high SES group manifested a higher number of colorectal cancers compared with low SES group (24.3% vs 19.0%, *P* < 0.001). Gastric, lung, liver, head and neck, gall bladder/biliary cancers were more frequently observed in low rather than in intermediate or high SES groups of patients.

**Table 1:** Patient characteristics

Variables	Low SES		Middle SES		High SES		*P*‐value
	N	%	N	%	N	%	
Age (y)							
60‐69	6970	28.4	14 463	27.9	8592	26.6	<0.001
70‐79	12 496	51.0	27 468	53.1	17 807	55.1	
≥80	5064	20.6	9839	19.0	5927	18.3	
Gender							
Male	11 899	48.5	25 964	50.2	16 780	51.9	<0.001
Female	12 631	51.5	25 806	49.8	15 546	48.1	
Insurance type							
Local	10 104	41.2	15 503	29.9	7929	24.5	<0.001
Workplace	9407	38.3	30 660	59.2	24 397	75.5	
Medical aid	5019	20.5	5607	10.8	0	0.0	
Disability							
No	24 248	98.9	51 161	98.8	32 012	99.0	<0.001
Yes	282	1.1	609	1.2	314	1.0	
CCI scores							
≤1	6865	28.0	14 156	27.3	8196	25.4	<0.001
>1	17 665	72.0	37 614	72.7	24 130	74.6	
Cancer diagnosis year							
2003‐2005	7109	29.0	15 155	29.3	9002	27.8	<0.001
2006‐2010	10 287	41.9	21 185	40.9	13 792	42.7	
2011‐2015	7134	29.1	15 430	29.8	9532	29.5	
Cancer site							
Colorectal	4668	19.0	11 459	22.1	7871	24.3	<0.001
Gastric	4617	18.8	9758	18.8	5935	18.4	
Lung	3910	15.9	7527	14.5	4297	13.3	
Liver	3386	13.8	6811	13.2	3971	12.3	
Head and neck	2532	10.3	4847	9.4	2725	8.4	
Urinary	1078	4.4	2690	5.2	1949	6.0	
Pancreas	957	3.9	1930	3.7	1345	4.2	
GB or biliary	685	2.8	1305	2.5	753	2.3	
Skin	539	2.2	1068	2.1	684	2.1	
Thyroid	497	2.0	1141	2.2	812	2.5	
Esophagus	465	1.9	840	1.6	471	1.5	
CNS	259	1.1	470	0.9	294	0.9	
Others	136	0.6	294	0.6	193	0.6	
Small intestine	129	0.5	243	0.5	148	0.5	
Bone or cartilage	111	0.5	239	0.5	127	0.4	
Multiple myeloma	101	0.4	174	0.3	128	0.4	
Non‐Hodgkin lymphoma	101	0.4	207	0.4	164	0.5	
Melanoma	100	0.4	214	0.4	131	0.4	
Connective tissue	99	0.4	239	0.5	120	0.4	
Leukemia	82	0.3	174	0.3	119	0.4	
Anus	78	0.3	140	0.3	89	0.3	
Cost per day (total mean, ₩)	154 385	163 257	169 319	<0.001			
Total	24 530	100.0	51 770	100.0	32 326	100.0	

Abbreviations: CCI, Charlson comorbidity index; CDI, composite deprivation index.

### 3.2 Socioeconomic status and overall/cancer‐specific survival

**Figure 2:**
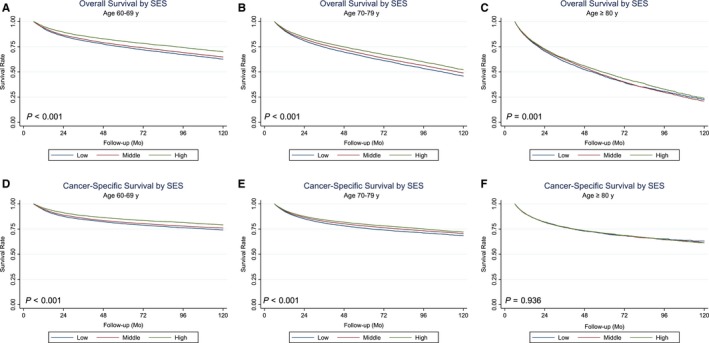
Kaplan‐Meier curves for overall survival according to SES for patients 60‐65 y old (A), 70‐79 y old (B), and ≥85 y old (C). Kaplan‐Meier curves for cancer‐specific survival according to SES are shown for patients 60‐65 y old (D), 70‐79 y old (E), and ≥85 y old (F). *P*‐values were estimated by the log‐rank test. SES, socioeconomic status

In univariate analysis, diagnosis year, age, gender, insurance type, the presence of disability, CCI score and SES levels were significant prognostic factors for OS and CSS (Table 2). In Kaplan‐Meier plots, the OS and CSS rates decreased according to SES levels (high to low) (Figure S2A,B). Also, advanced age (≥80 years) was the most hazardous factor in univariate analysis for OS (HR = 3.19, 95% CI: 3.10‐3.28, *P* < 0.001) and for CSS (HR 1.81, 95% CI 1.74‐1.88, *P* < 0.001), respectively. Then, we produced Kaplan‐Meier curves for OS and CSS according to SES, stratified by the three age groups: 60‐69, 70‐79, and ≥80 years. There were significant differences in OS according to SES in patients aged 60‐69 years (*P* < 0.001) (Figure 1A), 70‐79 years (*P* < 0.001) (Figure 1B), and ≥80 years (*P* < 0.001) (Figure 1C) and poorer survival outcomes were associated with lower SES. This trend was also found in CSS according to SES, particularly for patients 60‐69 (*P* < 0.001) (Figure 2D) and 70‐79 years old (*P* < 0.001) (Figure 2E). However, there was no significant difference in CSS by SES in patients more than 80 years old (*P* = 0.936) (Figure 2F).

**Table 2:** Univariate analysis of overall survival and cancer‐specific survival

	Overall survival			Cancer specific survival		
	HR	95% CI	*P*	HR	95% CI	*P*
Diagnosis year						
2003‐2005	1.00					
2006‐2010	1.10	1.07‐1.12	<0.001	1.05	1.02‐1.64	0.001
2011‐2015	1.16	1.12‐1.19	<0.001	1.08	1.04‐1.21	<0.001
Age (y)						
60‐69	1.00			1.00		
70‐79	1.64	1.60‐1.68	<0.001	1.29	1.25‐1.33	<0.001
≥80	3.19	3.10‐3.28	<0.001	1.81	1.74‐1.88	<0.001
Gender						
Male	1.00			1.00		
Female	0.63	0.62‐0.65	<0.001	0.53	0.51‐0.54	<0.001
Insurance type						
Local	1.00			1.00		
Workplace	0.96	0.94‐0.98	<0.001	0.97	0.95‐1.00	0.048
Medical aid	1.27	1.23‐1.31	<0.001	1.05	1.00‐1.10	0.039
Disability						
No	1.00			1.00		
Yes	1.80	1.67‐1.94	<0.001	1.28	1.14‐1.44	<0.001
CCI score						
≤1				1.00		
>1	1.09	1.07‐1.11	<0.001	0.87	0.85‐0.90	<0.001
SES level						
Low	1.08	1.05‐1.10	<0.001	1.08	1.04‐1.11	<0.001
Middle	1.00			1.00		
High	0.89	0.87‐0.91	<0.001	0.92	0.89‐0.94	<0.001

Abbreviations: CCI, Charlson Comorbidity Index; CI, confidence interval; HR, hazard ratio; SES, socioeconomic status.

*P* values were calculated from the Cox proportional hazard model.

**Figure 3:**
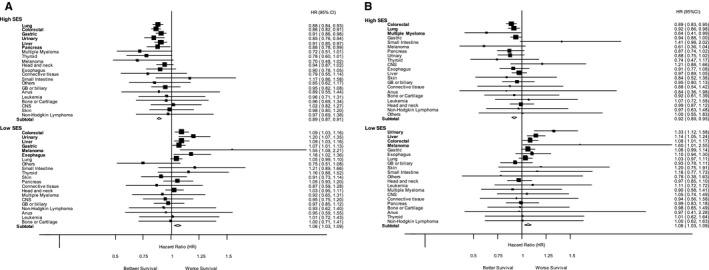
Subgroup analysis by primary cancer site. HR and 95% CI were calculated for overall survival (A) and cancer‐specific survival (B) in all study populations. *P*‐values were estimated by the Cox proportional hazard model using middle SES as the reference. CI, confidence interval; HR, hazard ratio; SES, socioeconomic status

According to cancer sites, we developed a forest plot showing each HR for OS (Figure 3A) and for CSS (Figure 3B). Patients with high SES diagnosed with lung, colorectal, gastric, urinary, liver, and pancreatic cancers showed better OS compared to middle SES patients with these cancers. Meanwhile, patients with low SES and diagnosed with colorectal, urinary, liver, gastric, melanoma, and esophageal cancers demonstrated worse OS. In terms of CSS, high SES along with colorectal, lung, and multiple myeloma resulted in better survival and low SES combined with urinary, liver, and colorectal cancers, and melanoma demonstrated worse survival compared with those in intermediate SES, respectively.

Next, we performed multivariate analysis for OS (Table 3) and CSS (Table 4) stratified by age group, adjusting for diagnosis year as a time‐dependent variable. High SES was a favorable prognostic factor for OS in patients aged 60‐69 years (HR = 0.85, 95% CI: 0.81‐0.89, *P* < 0.001), 70‐79 years (HR = 0.90, 95% CI: 0.87‐0.93, *P* < 0.001), and ≥80 years (HR = 0.91, 95% CI: 0.87‐0.96, *P* < 0.001). However, in patients 70‐79 years old, low SES was a significantly unfavorable prognostic factor for OS (HR = 1.08, 95% CI: 1.05‐1.12, *P* < 0.001) (Table 3) and CSS (HR = 1.11, 95% CI: 1.06‐1.16, *P* < 0.001) (Table 4). High SES was not associated with CSS in patients ≥80 years old (*P* = 0.101) but was significantly associated with favorable CSS in those aged 60‐69 (HR = 0.87, 95% CI: 0.82‐0.92, *P < *0.001) and in those aged 70‐79 (HR = 0.92, 95% CI: 0.88‐0.96, *P* < 0.001). Both female and recently diagnosed year were consistently favorable factors for OS and CSS across all age groups (All *P* < 0.001).

**Table 3:** Multivariate analyses of overall survival stratified by age

	Multivariate (60‐69 y)			Multivariate (70‐79 y)			Multivariate (≥80 y)		
	HR	95% CI	*P*	HR	95% CI	*P*	HR	95% CI	*P*
Gender									
Male	1.00			1.00			1.00		
Female	0.47	0.45‐0.49	<0.001	0.56	0.55‐0.58	<0.001	0.69	0.67‐0.72	<0.001
Insurance type									
Local	1.00			1.00			1.00		
Workplace	0.94	0.91‐0.98	0.007	0.96	0.93‐0.99	0.010	0.99	0.95‐1.04	0.802
Medical aid	1.46	1.35‐1.58	<0.001	1.17	1.12‐1.23	<0.001	1.05	0.99‐1.12	0.123
Disability									
No	1.00			1.00			1.00		
Yes	1.48	1.21‐1.81	<0.001	1.42	1.27‐1.58	<0.001	1.24	1.10‐1.39	<0.001
CCI scores									
≤1	1.00			1.00			1.00		
>1	1.12	1.07‐1.17	<0.001	1.10	1.06‐1.13	<0.001	1.01	0.97‐1.06	0.562
SES level									
Low	1.04	0.99‐1.09	0.093	1.08	1.05‐1.12	<0.001	1.03	0.98‐1.08	0.304
Middle	1.00			1.00			1.00		
High	0.85	0.81‐0.89	<0.001	0.90	0.87‐0.93	<0.001	0.91	0.87‐0.96	<0.001
Diagnosis year^a^	1.00	1.00‐1.00	0.002	1.00	1.00‐1.00	<0.001	1.00	1.00‐1.00	<0.001

Abbreviations: CCI, Charlson comorbidity index; CI, confidence interval; HR, hazard ratio; SES, socioeconomic status.

a Time‐dependent continuous variable. Actual HRs and CIs at “Diagnosis year” row ranged from 0.999 to <1.000.

**Table 4:** Multivariate analyses of cancer‐specific survival stratified by age

	Multivariate (60‐69 y)			Multivariate (70‐79 y)			Multivariate (≥80 y)		
	HR	95% CI	*P*	HR	95% CI	*P*	HR	95% CI	*P*
Gender									
Male	1.00			1.00			1.00		
Female	0.44	0.42‐0.46	<0.001	0.50	0.49‐0.52	<0.001	0.58	0.55‐0.61	<0.001
Insurance type									
Local	1.00			1.00			1.00		
Workplace	0.96	0.91‐1.01	0.110	0.97	0.93‐1.01	0.150	1.02	0.95‐1.08	0.577
Medical aid	1.26	1.13‐1.39	<0.001	1.02	0.96‐1.08	0.538	0.94	0.85‐1.02	0.174
Disability									
No	1.00			1.00			1.00		
Yes	1.06	0.80‐1.41	0.681	1.16	0.99‐1.35	0.072	0.93	0.77‐1.12	0.432
CCI score									
≤1	1.00			1.00			1.00		
>1	0.89	0.85‐0.94	<0.001	0.89	0.85‐0.93	<0.001	0.81	0.76‐0.87	<0.001
SES level									
Low	1.06	1.00‐1.12	0.052	1.11	1.06‐1.16	<0.001	1.03	0.96‐1.10	0.454
Middle	1.00			1.00			1.00		
High	0.87	0.82‐0.92	<0.001	0.92	0.88‐0.96	<0.001	0.95	0.88‐1.01	0.101
Diagnosis year^a^	1.00	1.00‐1.00	0.002	1.00	1.00‐1.00	<0.001	1.00	1.00‐1.00	<0.001

Abbreviations: CCI, Charlson comorbidity index; CI, confidence interval; HR, hazard ratio; SES, socioeconomic status.

a Time‐dependent continuous variable. Actual HRs and CIs at “Diagnosis year” row ranged from 0.999 to <1.000.

## 4 DISCUSSION

The current study revealed that SES as defined by household income and degree of regional deprivation was a significant prognostic indicator of OS and CSS in the NHISS‐ESC database. It is noteworthy that patients with high SES living in an advantageous region and earning high income showed better OS, regardless of age, and better CSS, except in participants ≥80 years old, compared with patients with low SES. The survival disparities remained significant even after adjusting for gender, insurance type, the presence of disability, comorbidities, and diagnosis year as a time‐dependent variable that are important for survival in elderly patients.

Numerous studies reported that SES mainly derived from income correlated negatively with OS and CSS in elderly cancer patients.^13^, ^14^, ^15^ However, previous studies have reported that a person's income is not fully representative of the individual SES level,^16^, ^17^ which is commonly explored in studies investigating cancer health disparities. On the basis of previous findings,^18^, ^19^, ^20^ neighborhood poverty is considered one of the factors contributing to cancer mortality as well. However, the impact of regional SES on CSS was not consistent among studies.^8^ In current study, both individual income and regional status were considered to define SES. Because most of SES studies were based on multi‐ethnic countries such as the United States, race or ethnicity per se might be a major determining factor underlying individual SES. But, our work was based on a national database derived from a single Asian ethnic country, and therefore, ethnic disparities are not a confounding factor in SES. A more recent diagnosis year might be related to better treatment, favoring overall survival. Thus, we considered diagnosis year as a time‐dependent variable in Cox hazard proportional analysis, given the long study period (2003‐2015). Collectively, we observed an association between SES and OS or CSS in elderly cancer patients.

Even though the financial or knowledge barriers separating cancer patients from access to health‐care resources are decreasing currently in Korea, the stratification of SES by income and regional deprivation could extend our understanding of its impact on CSS as well as OS for elderly patients with cancer. However, these findings were no longer significant difference in CSS according to SES for patients with advanced age (≥80 years) due to several possible reasons. For instance, most of them retire from their jobs, and other family members are likely to pay their health insurance premiums instead. Also, even when classified into a low‐income group, much of their accumulated property is already likely to be inherited by their children. Their knowledge, understanding of their disease, prestige, or social connections depending on residence^21^ must have declined with advanced age. Thus, among the very elderly cancer patients, strategies other than alleviation of SES disparities should be considered.

Subgroup analysis by cancer sites showed the existence of specific cancer categories, which were affected significantly by high or low SES. Overall, survival differences depend on high vs low SES. In terms of OS, elderly patients with lung cancer were found to derive the most benefit according to their high SES, and those with colorectal cancer were the most disadvantaged by their low SES. On the other hand, in terms of CSS, colorectal cancer patients with high SES and urinary cancer patients with low SES were most affected by their SES, respectively. Because few studies have examined the relationship between survival and SES for different types of cancer, further studies are needed to investigate the detailed mechanism underlying these observations, suggesting that different approaches are necessary to mitigate SES disparities in patients within each category of cancer.

More importantly, CSS was also dependent on SES among elderly cancer patients. As expected, elderly patients usually exhibit one or more comorbid geriatric syndromes, which affect their OS.^22^ Aside from comorbidities, however, we observed differences in CSS according to SES, which can be attributed to several possible factors. Cancer stage and the subsequent oncologic outcome can be attributed to several factors of SES. A Danish study found that the advanced stage is related to low SES in lymphoma.^23^ Consistent with this finding, in solid cancers, the incidence of metastatic disease is significantly higher in unmarried patients, emphasizing social support.^24^ The relationship between SES and cancer burden may lead to aggressive policy measures and interventions in elderly patients with low SES. However, this relationship was not confirmed in the current study due to the paucity of information about cancer tumor burden such as cancer stage.

Not all costs related to cancer diagnosis and treatment are covered by the Korean government. There are out‐of‐pocket expenditures for charges not covered by health insurance, which are an economic burden to individual patients.^25^, ^26^, ^27^ Also, cancer centers with expert oncologists are not evenly distributed across the country but are concentrated in some megacities. Limited access to cancer‐screening programs, advice from social connections, or hospital visits, as well as financial circumstances^28^, ^29^, ^30^ may lead to the prevalence of advanced cancer among elderly patients with low SES. Thus, we speculated that the out‐of‐pocket expenditures, initial access to the health‐care system, frequent visits to cancer centers, hospitalization for surgery, weekly based chemotherapy, or daily based radiation therapy, depended on individual SES factors, such as income, knowledge, employment status, housing, and social network.

Our findings remind of the importance of SES in oncologic survival. However, there are several limitations. The database used in the current study masks major cancer types including breast, prostate, gynecological, or male/female genital cancers, preventing analysis of patients with these cancers. Further, as mentioned earlier, the disease information such as tumor stage could not be identified in the database. Lower SES might be linked with more advanced tumor stages, resulting in poor OS or CSS compared to patients with higher SES. However, this association or causal relationship could not be addressed in the current study because tumor stage information was not collected. Nevertheless, the strength of our study is that we used an unprecedentedly large elderly national cohort data with accurate information that was not derived from patients or researchers but rather from a national system with built‐in quality assurance, suggesting data integrity and minimal variability. Finally, our conclusions may not be applicable to other countries due to different health‐care systems, local culture, and medical practice guidelines.

Taken together, the SES determined by household income and degree of regional deprivation is a significant factor in the survival of elderly cancer patients. Subgroup analysis based on primary cancer sites revealed the impact of low or high SES on specific cancers. The underlying mechanism such as the association between SES and tumor burden warrants further investigation. A public health strategy with high precision needs to be established to target groups stratified by age and cancer sites.

## CONFLICT OF INTEREST

The authors of this article declared they have no conflicts of interest.
