# Supplementary figures and images for: Socioeconomic status and survival outcomes in elderly cancer patients: A national health insurance service‐elderly sample cohort study
Source: Cancer Med. 2019 May 8;8(7):3604–13. doi: 10.1002/cam4.2231 (PMC6601595; doi:10.1002/cam4.2231)

(A) Log-Log plot according to SES for OS

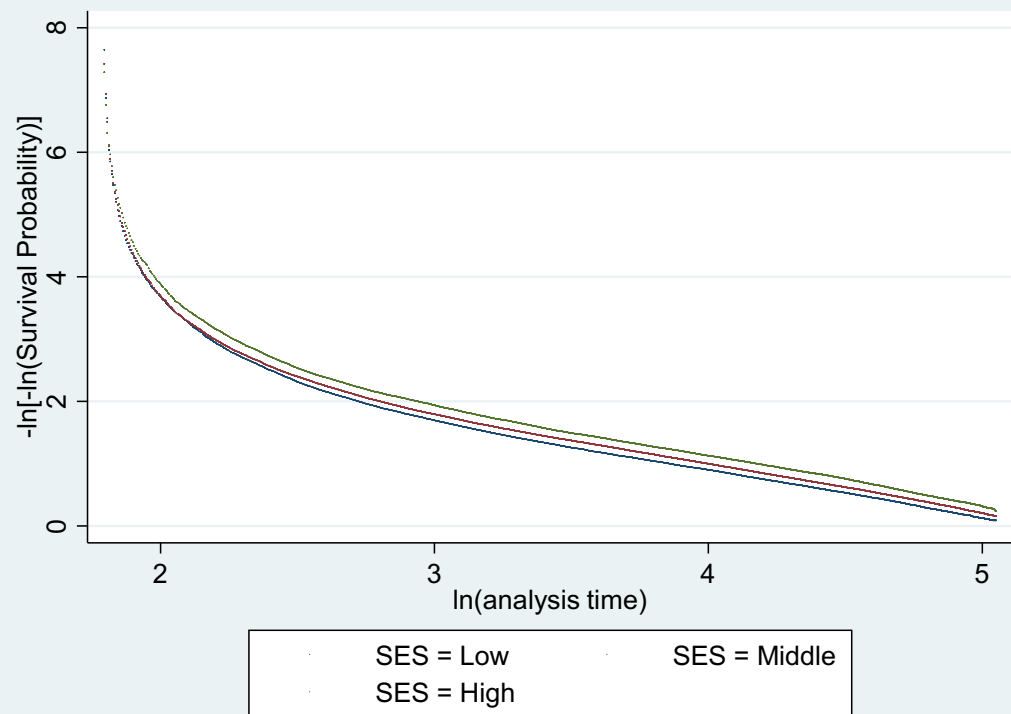

(B) Log-Log plot according to SES for CSS

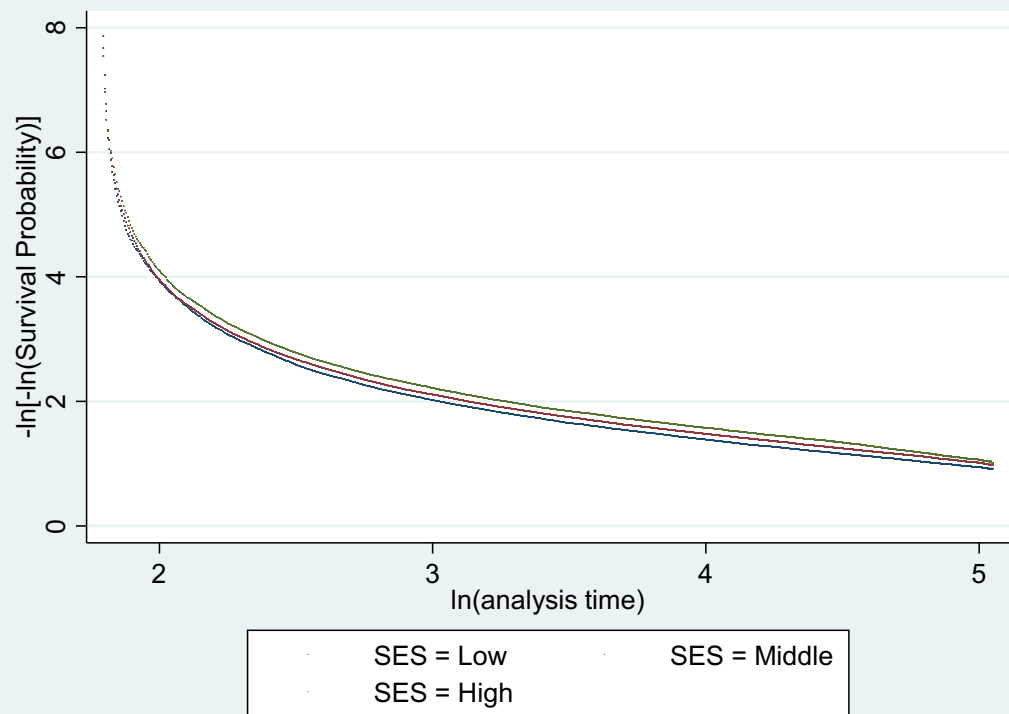

Supplement: Supplementary file 1 [file CAM4-8-3604-s001.pdf]

(A)

Overall Survival by SES

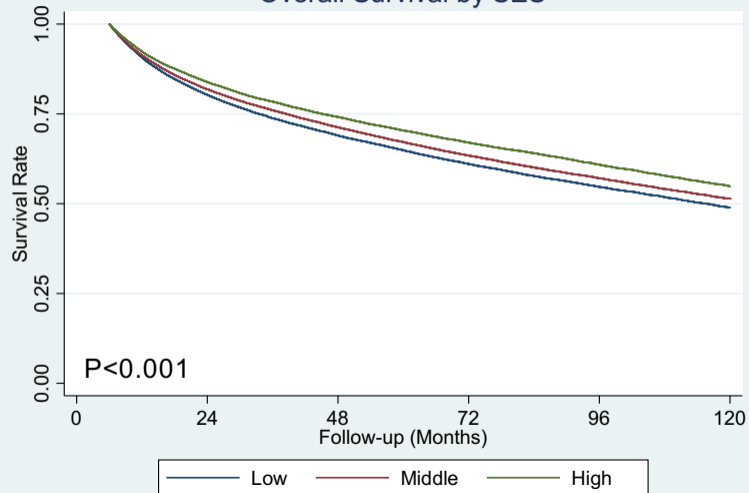

(B)

Cancer-Specific Survival by SES

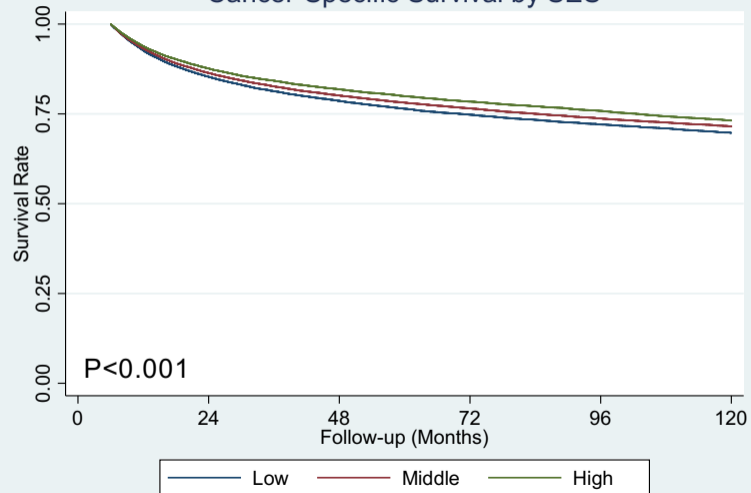

Supplement: Supplementary file 2 [file CAM4-8-3604-s002.pdf]
